# Guanylate-binding protein 1 participates in cellular antiviral response to dengue virus

**DOI:** 10.1186/1743-422X-9-292

**Published:** 2012-11-27

**Authors:** Wen Pan, Xiangyang Zuo, Tingting Feng, Xiaohong Shi, Jianfeng Dai

**Affiliations:** 1 Institute of Biology and Medical Sciences, Jiangsu Key Laboratory of Infection and Immunity, Soochow University, Building 703, 199 Ren-ai Road, Suzhou, 215123, P.R. China

**Keywords:** GBP1, DENV, Antiviral response, NF-κB

## Abstract

**Background:**

Dengue virus (DENV), the causative agent of human Dengue hemorrhagic fever, is a mosquito-borne virus found in tropical and sub-tropical regions around the world. Vaccines against DENV are currently unavailable. Guanylate-binding protein 1 (GBP1) is one of the Interferon (IFN) stimulated genes (ISGs) and has been shown important for host immune defense against various pathogens. However, the role of GBP1 during DENV infection remains unclarified. In this study, we evaluated the relevance of GBP1 to DENV infection in *in vitro* model.

**Findings:**

Quantitative RT-PCR (qRT-PCR) and Western blot showed that the expression of mouse *Gbp1* was dramatically upregulated in DENV-infected RAW264.7 cells. The intracellular DENV loads were significantly higher in *Gbp1* silenced cells compared with controls. The expression levels of selective anti-viral cytokines were decreased in *Gbp1* siRNA treated cells, while the transcription factor activity of NF-κB was impaired upon *GBP1* silencing during infection.

**Conclusions:**

Our data suggested that GBP1 plays an antiviral role during DENV infection.

## Findings

Dengue virus (DENV), a member of the mosquito-borne flavivirus family, is an icosahedral, enveloped virus with a single-stranded positive sense RNA genome. Millions of cases of DENV infection occur worldwide each year [1,2]. Dengue hemorrhagic fever, the severe form of DENV infections, can cause serious haemorrhage, sudden drop in blood pressure (shock) and even death. Since no vaccine against DENV is currently available, much effort is needed to explore the host antiviral mechanisms for control of DENV infection and vaccine development [1,3-5].

Interferons (IFNs) are major antiviral cytokines released by host cells in response to viral and other pathogenic infections and play crucial roles in induction and regulation of both innate and adaptive immune responses [6]. IFNs establish an antiviral state by activating mainly the Janus kinase/signal transducer and activator of transcription (JAK/STAT) signaling pathway and other signaling cascades. Even though hundreds of genes are characterized as interferon-stimulated genes (ISGs), the roles of many ISGs during infection remain largely unknown [7,8].

Guanylate-binding protein 1 (GBP1) is one of the ISGs that most strongly induced by IFNs [9] and belongs to a family of GTPases which are divided into three groups: (1) the large GTPases, also known as GBPs; (2) the small GTPases; (3) the Mx proteins. The human large GTPase family is composed of seven members encoded by a gene cluster located on chromosome 1 [10,11]. The emerging roles of GBP1 in host immune responses have been characterized in *in vitro* and *in vivo* models [12-15]. For example, GBP1 is overexpressed in endothelial cells upon activation of inflammatory cytokines and is involved in intestinal mucosal inflammation [16]. Recently, a study using *Gbp1* knockout mice indicated that GBP1 has strong anti-bacterial activity [17]. There are also reports suggesting that GBP1 is involved in host immune response against chlamydia [18] and viruses including vesicular stomatitis virus, encephalomyocarditis virus and Hepatitis C Virus [19-21]. However, the distinct role of GBP1 during infection of other pathogens, including mosquito-borne flaviviruses remains largely unknown. We hereby investigated the physiological role of GBP1 during DENV infection in different *in vitro* models.

### *Gbp1* is upregulated upon DENV infection

**Figure 1:**
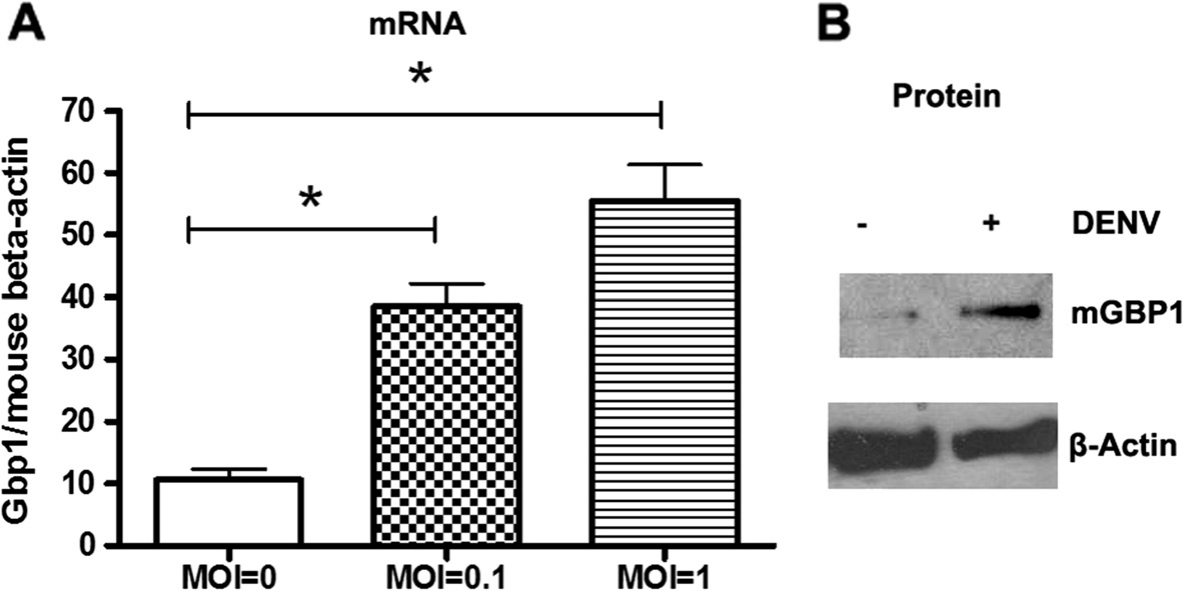
***Gbp1 *is upregulated upon DENV infection.A**. The gene expression was analyzed by quantitative RT-PCR (qRT-PCR), and normalized to mouse beta actin gene. Results are expressed as the mean + the SEM. * *p* < 0.05 (*t-*test). **B**. The protein level of mouse GBP1 was increased in DENV infected cells compared with uninfected cells, as shown in Western blot using anti- GBP1 antibody (sc 28579, Santa Cruz, CA). Representative results from at least 3 independent experiments.

Mouse macrophage cell line RAW264.7 can be readily infected by DENV, and served as an *in vitro* model for the study of host innate immune response to DENV. By using a quantitative RT-PCR (qRT-PCR) based small cDNA array (SABiosciences, Frederick, MD), we measured the expression profile of genes from Jak-Stat signaling pathway in DENV (DENV 2, New Guinea C strain) infected RAW264.7 cells. A number of genes were found up- or down-regulated upon DENV infection, as reported in our previous work (Additional file 1of Ref. [22]). Among them, *Gbp1* was upregulated 3.97-fold in DENV infected cells in comparison to uninfected controls. Independent qRT-PCR and Western blotting further confirmed the upregulation of GBP1 (mRNA and protein) upon DENV infection (Figure 1, A and B). Recent studies have identified host genes that upregulated in human patients with acute DENV infection [23,24]. *GBP1* was one of the genes most highly induced in patients’ blood cells during early DENV infection [23], and this is consistent with our result here shown in *in vitro* study.

### *Gbp1* has antiviral activity against DENV infection

**Figure 2:**
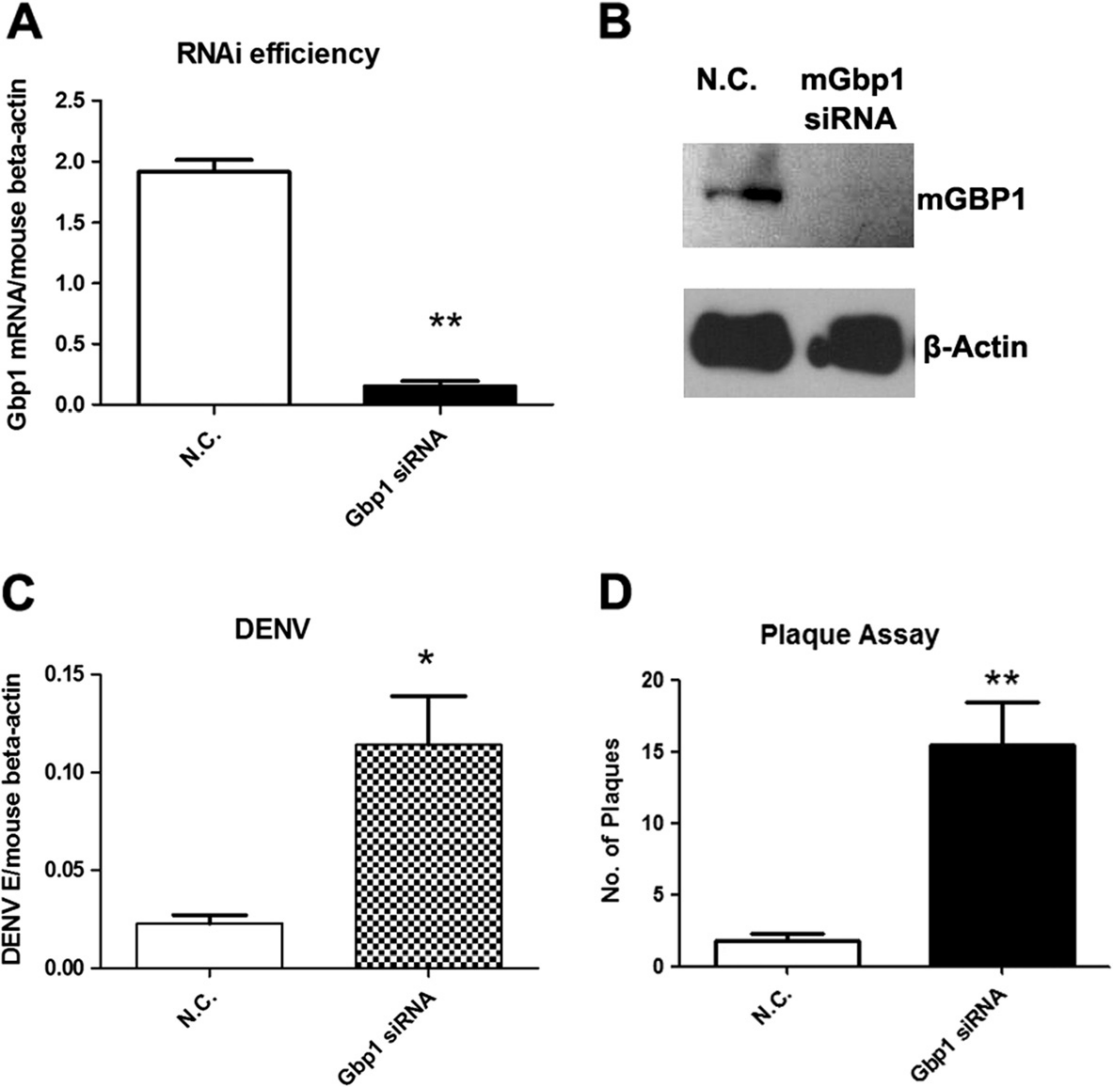
**The viral burden is increased in *Gbp1 *silenced cells.A**-**B**) RNAi efficiency for *Gbp1* as shown in qRT-PCR (**A**) and Western blot (**B**). **C**-**D**) DENV burdens in RAW264.7 cells after RNAi silencing. **C**) The viral burdens were analyzed by measuring the virus E gene copy using qRT-PCR, and normalized to mouse beta actin gene. **D**) The titer of infectious DENV in supernatants of cells. Y axis represents the number of plaques formed in 293T cells when infected with viruses in 100 μl of cell supernatants 24h post infection. Results are expressed as the mean + the SEM. * *p* < 0.05 and ** *p* < 0.01 (*t-*test). Representative results from at least 3 independent experiments.

To study the specific role of GBP1 during DENV infection, an siRNA based RNA interference study was performed in RAW264.7 cells. siRNAs specific for *Gbp1* or scrambled control (N.C.) were delivered into RAW264.7 cells respectively via electroporation. 24hrs after siRNA transfection, cells were challenged by DENV (MOI=1.0) for another 24hrs. Cells were then harvested and total RNA and cDNA were made according to standard protocols. *Gbp1* was silenced efficiently as analyzed by qRT-PCR (Figure 2A) using gene specific primers (Table 1) and by Western blot (Figure 2B). The intracellular viral loads, in terms of the transcript levels of the envelop gene (E), were quantified by qRT-PCR and normalized to mouse beta-actin gene. As shown in Figure 2C, the DENV viral load was increased 5.0-fold (*p*<0.05) in *Gbp1* silenced cells compared with control cells. To measure the production of infectious virus from these cells, a plaque assay was performed in 293T cells. The titers of virus in supernatants from *Gbp1* silenced cells were about 10- fold higher compared with that from control cells (Figure 2D). These data suggested that *Gbp1* has an anti-viral activity against DENV in RAW264.7 cells.

**Table 1:** siRNA and oligo-primer sequences for this study

**No.**	**Sequence (5’-3’)**	**Note**
1	GGAACGUAUAAAAGCAGAAtt	siRNA seq for mouse gene *Gbp1*
2	AAGGCAUGUACCAUAAGCUtt	siRNA seq for human gene *GBP1*
3	AGAGGGAAATCGTGCGTGAC	Forward primer for qRT-PCR of mouse beta-actin
4	CAATAGTGATGACCTGGCCGT	Reverse primer for qRT-PCR of mouse beta-actin
5	GGGCATGGAGTCCTGTGGCA	Forward primer for qRT-PCR of human beta-actin
6	GGGTGCCAGGGCAGTGATCTC	Reverse primer for qRT-PCR of human beta-actin
7	CATTCCAAGTGAGAATCTCTTTGTCA	Forward primer for qRT-PCR of DENV E gene
8	CAGATCTCTGATGAATAACCAACG	Reverse primer for qRT-PCR of DENV E gene
9	GGGCAGCTGTCTTTGGGTAGAC	Forward primer for qRT-PCR of mouse *Gbp1* gene
10	AGCATGAGGCCCTAGGAGCTGT	Reverse primer for qRT-PCR of mouse *Gbp1* gene
11	AAGAGAGGACCCTCGCTCTTA	Forward primer for qRT-PCR of human *GBP1* gene
12	ATGCCTTGGTTAGGGGTGAC	Reverse primer for qRT-PCR of human *GBP1* gene

### Selective cytokines are downregulated in *Gbp1* silenced cells upon DENV infection

**Figure 3:**
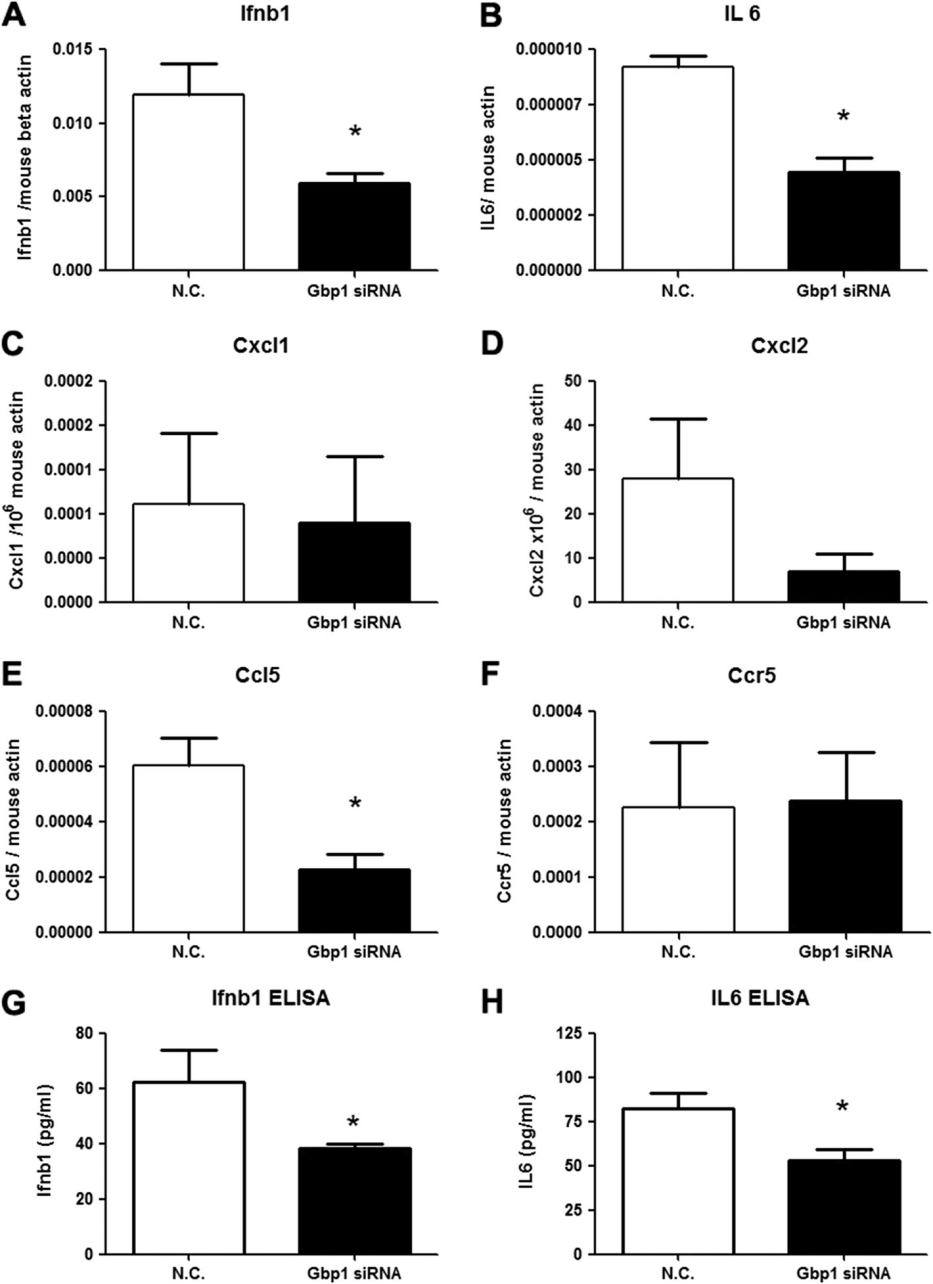
**Cytokine expression in DENV infected cells after gene silencing.A**-**F**) mRNA levels of selective cytokines/chemokines (A:*Ifnβ1*, B:*Il 6*, C:*Cxcl1*,D:*Cxcl2*,E:*Ccl5*,F:*Ccr5*) were measured by qRT-PCR and normalized to mouse beta-actin gene. **G**-**H**) Protein level of IFNβ and IL6 in cell supernatants. Results are expressed as the mean + the SEM. * *p* <0.05 (*t-*test). Representative results from at least 3 independent experiments.

In order to examine whether the silencing of *Gbp1* would affect the expression of cytokines, the expressions of some representative cytokines and chemokines were measured in *Gbp1* siRNA or scrambled siRNA treated cells after DENV infection. Total of six genes were chosen for the study, including type I interferon (*Ifnβ1*), Interleukin-6 (*Il6*), Chemokine (C-X-C motif) ligand 1 (*Cxcl1*), Chemokine (C-X-C motif) ligand 2(*Cxcl2*), Chemokine (C-C motif) ligand 5(*Ccl5*) and C-C chemokine receptor type 5(*Ccr5*). Gene specific primers and probe sets were purchased from Applied Biosystems (Carlsbad, CA). After DENV infection, the transcription levels of *Ifnβ1, Il6,* and *Ccl5* in *Gbp1* silenced groups were decreased 2 to 3-fold comparing with controls (*p*<0.05). However, transcription levels of *Cxcl1/2* and *Ccr5* were not significantly affected (Figure 3, A-F)*.* Consistent with the mRNA expression, the protein secretion of IFNβ and IL6 were decreased in *Gbp1* silenced cells (Figure 3, G and H). These data suggested that antiviral role of *Gbp1* is associated with its ability to modulate some antiviral or pro-inflammatory cytokines during DENV infection.

### Transcriptional factor activity of NF-ÎºB is impaired in *GBP1* silenced cells

**Figure 4:**
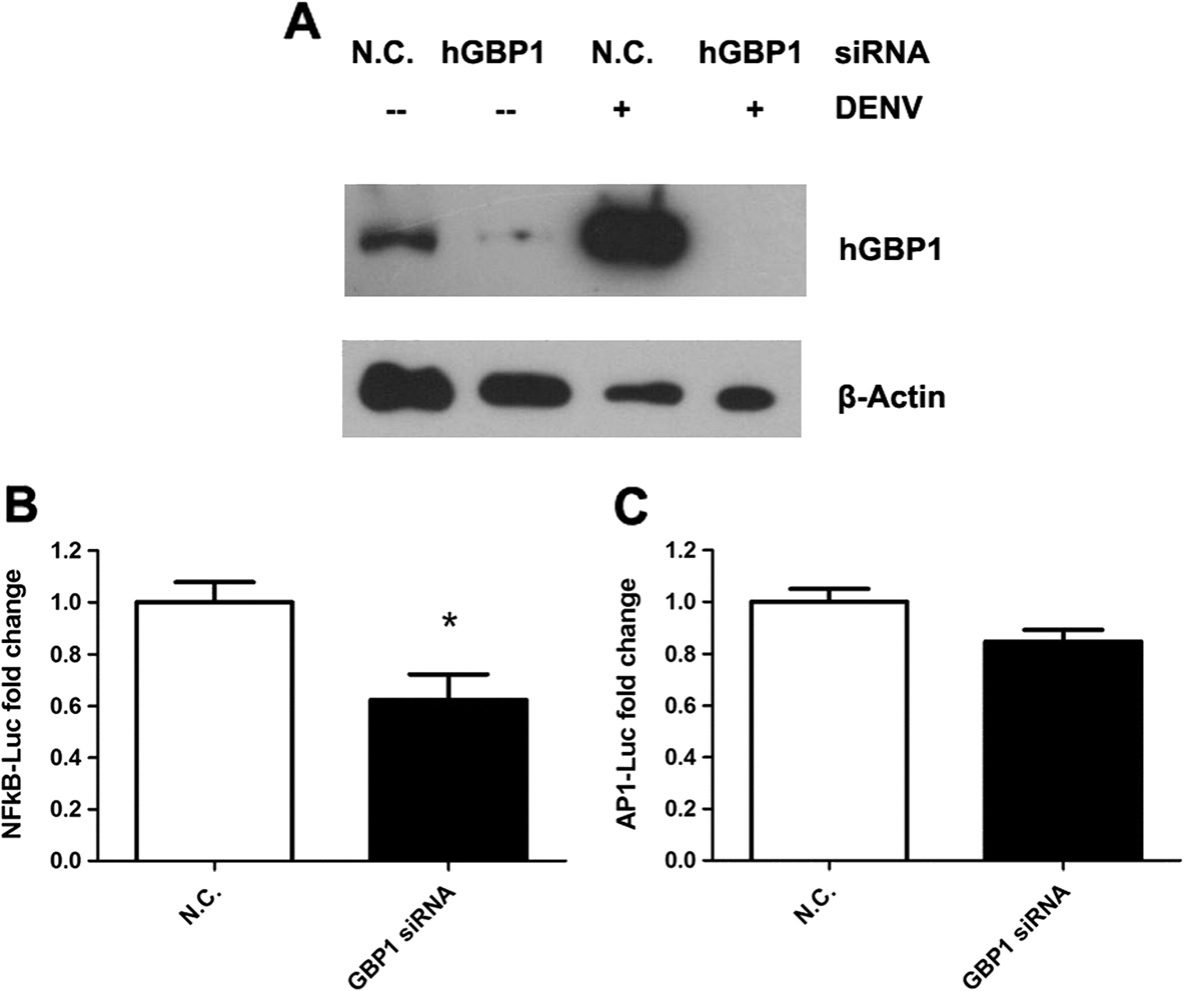
**NF-κB activity is impaired upon *GBP1 *silencing.** (**A**) GBP1 was silenced efficiently in 293T cells as shown in western blot. NF-κB (**B**) and AP1 (**C**) activities were measured by dual luciferase reporter assay. (The reporter activities were normalized by internal control (pRL-TK Renilla luciferase value). The mean value of activities from control cells were set to 1.0) Results are expressed as the mean + the SEM. * *p* < 0.05 (*t-*test). Representative results from at least 3 independent experiments.

NF-κB (nuclear factor- kappa B) and AP1 (activator protein 1) play crucial roles in antiviral innate immune response through promoting the transcription of numerous antiviral or pro-inflammatory genes [25,26]. Since the expression levels of some cytokines were decreased in *Gbp1* silenced cells after DENV infection, we further measured the activities of NF-κB and AP1 upon GBP1 silencing. 293 cells can be robustly infected by DENV[27], and are the most common system to measure the transcriptional factor activity using dual luciferase reporter assay. Reporter plasmids pGL- NF-κB, pGL-AP1 and pRL-TK (internal control) (Promega, Madison, MI) in together with siRNAs specific for human *GBP1* or scrambled control (N.C.), were delivered into 293T cells respectively by transfection using Lipofectamine® LTX & PLUS (Invitrogen, Grand Island, NY). 24hrs after transfection, cells were challenged by DENV (MOI=1.0) for another 24hrs, respectively. RNAi efficiency was confirmed by Western blot (Figure 4A). Cells were then harvested and lysed for dual luciferase reporter assay (Promega, Madison, MI). The relative activity of NF-κB was decreased by 40% in *GBP1* knockdown cells in comparison with controls; while the AP1 activity was not significantly impaired in this case (Figure 4, B and C). These data suggested that GBP1 may influence activity of NF-κB, thereby contribute to the production of anti-viral or pro-inflammatory cytokines/chemokines. This could also partially address the mechanisms of how GBP1 inhibits DENV replication in cell culture.

More and more attention has been focused on the roles of large GTPase family in immune response [12-14]. GBP1 can be induced by IFNγ as well as IFNα/β, and its induction can be augmented by TNF-α, IL-1 or LPS[12]. The inhibitory roles of GBP1 to different pathogens have been reported [17-19,21]. Itsui and his colleague showed that HCV replication was suppressed significantly by overexpression of GBP1, while binding of the HCV-NS5B protein to GBP1 countered the antiviral effect through inhibition of GTPase activity [21]. MacMicking speculated that GBP1 might help to limit the cell-to-cell spread of progeny virus through its anti-cell proliferative activity [13,28]. GBP1 has modest antiviral activity against the negative strand RNA viruses (such as Rhabdovirus, vesicular stomatitis virus), as well as the positive strand RNA viruses (Picornovirus and encephalomyocarditis virus) in cultured cells [19]. In both anti- chlamydia and antiviral study, the GTPase domain of GBP1 was suggested to be critical for its inhibitory activity [18,21]. A recent *in vivo* study further suggested that GBPs can solicit host defense proteins, including the phagocyte oxidase, antimicrobial peptides, and autophagy effectors, to kill intracellular bacteria [17]. While, more work are still needed to address the role and mechanisms of GBP1 in different infection models especially for viruses.

In this study, we confirmed that GBP1 shows inhibitory effect on DENV infection, influences the activity of NF-κB and further contributes to the production of anti-viral and pro-inflammatory cytokine/chemokines. This could be a novel mechanism for GBP1 to exert its antiviral activity in cellular level. Since NF-κB can be activated upon a broad range of pathogen infection, this could be a common action for GBP1 to play its role in different infection models. Further experiments will address the detail mechanisms of GBP1 on influencing NF-κB pathway in various models.

## Abbreviations

GBP1: Guanylate-binding protein-1; DENV: Dengue virus; Ifnβ1: Type I interferon; Il6: Interleukin-6; Cxcl1: Chemokine (C-X-C motif) ligand 1; Cxcl2: Chemokine (C-X-C motif) ligand 2; Ccl5: Chemokine (C-C motif) ligand 5; Ccr5: C-C chemokine receptor type 5; NF-κB: Nuclear factor kappa B; AP1: Activator protein 1.

## Competing interests

The authors declare that they have no competing interests.

## Authors contribution

JD, WP and XS designed the experiments and prepared the manuscript. XZ, WP and TF performed all the experiments. All authors read and approved the final manuscript.
